# Abdominal Imaging Utilization during the First COVID-19 Surge and Utility of Abdominal MRI

**DOI:** 10.3390/tomography7040080

**Published:** 2021-12-15

**Authors:** Mark A. Anderson, Reece J. Goiffon, Simon Lennartz, Rajesh Bhayana, Avinash Kambadakone

**Affiliations:** 1 Department of Radiology, Massachusetts General Hospital, Boston, MA 02114, USA; rgoiffon@mgh.harvard.edu (R.J.G.); simon.lennartz@uk-koeln.de (S.L.); akambadakone@mgh.harvard.edu (A.K.); 2 Faculty of Medicine, Institute for Diagnostic and Interventional Radiology, University Hospital Cologne, Kerpener Straße 62, 50937 Cologne, Germany; 3 Department of Medical Imaging, Toronto General Hospital, University of Toronto, Toronto, ON M5T 1W7, Canada; rajesh.bhayana@utoronto.ca

**Keywords:** SARS-CoV-2, liver function tests, radiography, computed tomography

## Abstract

We sought to determine relative utilization of abdominal imaging modalities in coronavirus disease 2019 (COVID-19) patients at a single institution during the first surge and evaluate whether abdominal magnetic resonance imaging (MRI) changed diagnosis and management. 1107 COVID-19 patients who had abdominal imaging were analyzed for modality and imaging setting. Patients who underwent abdominal MRI were reviewed to determine impact on management. Of 2259 examinations, 80% were inpatient, 14% were emergency, and 6% were outpatient consisting of 55% radiograph (XR), 31% computed tomography (CT), 13% ultrasound (US), and 0.6% MRI. Among 1107 patients, abdominal MRI was performed in 12 within 100 days of positive SARS-CoV-2 PCR. Indications were unrelated to COVID-19 in 75% while MRI was performed for workup of acute liver dysfunction in 25%. In 1 of 12 patients, MRI resulted in change to management unrelated to COVID-19 diagnosis. During the first surge of COVID-19 at one institution, the most common abdominal imaging examinations were radiographs and CT followed by ultrasound with the majority being performed as inpatients. Future COVID-19 surges may place disproportionate demands on inpatient abdominal radiography and CT resources. Abdominal MRI was rarely performed and did not lead to change in diagnosis or management related to COVID-19 but needs higher patient numbers for accurate assessment of utility.

## 1. Introduction

A number of gastrointestinal (GI) manifestations have been reported in patients with coronavirus disease 2019 (COVID-19), including ileus, Ogilvie syndrome, GI bleeding, bowel ischemia, liver function abnormalities, acute hepatitis, fulminant liver failure, cholestasis, acalculous cholecystitis, cholangitis, and pancreatitis [1,2,3,4,5,6,7,8,9,10,11,12,13]. Gastrointestinal symptoms are common, with meta-analyses of COVID-19 patients showing pooled incidence of any GI symptom in 12%, including abdominal pain in 4–5%, diarrhea in 7–8%, and nausea or vomiting in 5–8% [14,15]. At one institution, 45% of COVID-19 patients admitted to the surgical intensive care unit (ICU) experienced GI symptoms [3]. Some COVID-19 patients present with only GI manifestations, in the absence of respiratory symptoms [5,9,11,13,16]. Pathophysiologic support of GI organ involvement includes fecal shedding of the causative SARS-CoV-2 RNA in 40–50% of COVID-19 patients and epithelial expression of its host cell entry point, the angiotensin-converting enzyme 2 (ACE-2) receptor, in the esophagus, stomach, small bowel, colon, rectum, hepatocytes, cholangiocytes, and pancreatic islet cells [2,15,17,18,19].

Acute liver injury is common in patients with COVID-19, but is most often mild [4,7]. Elevations in aspartate transferase (AST) and alanine transferase (ALT), alkaline phosphatase (ALP) and gamma-glutamyltransferase (GGT), and hyperbilirubinemia occur in isolation or combination in COVID-19, indicating variable hepatocellular injury, cholestasis, and impaired biliary secretion [3,4,14,15]. Severe liver function test (LFT) abnormalities accompanying acute liver failure, liver ischemia and necrosis, and liver-related mortality in COVID-19 have been reported [3,8,9,20]. Among the <10% of COVID-19 patients with severe LFT abnormalities, prognosis is worse compared to COVID-19 patients without LFT abnormalities, including significantly higher rates of ICU admission, intubation, renal replacement therapy, and mortality [4,7]. Differentiation of liver function abnormalities due to COVID-19 versus from another cause is important, given both the prognostic implications for COVID-19 patients and because there are treatable non-COVID causes such as obstructive choledocholithiasis and portal vein thrombosis. The American Gastroenterology Association recommends checking baseline LFTs in all COVID-19 patients at admission, monitoring LFTs throughout their hospitalization, and to investigate non-COVID-19 causes of liver disease in all patients with abnormal LFTs [14]. What role imaging, particularly abdominal magnetic resonance imaging (MRI), should play in this workup has not been defined.

Abnormal computed tomography (CT) findings correlating with COVID-19 GI manifestations have been reported by multiple groups, including bowel abnormalities in up to 31% of inpatients imaged by CT, bowel ischemia in 20% of CTs in ICU patients, case reports of pancreatitis, and a single case of obstructive cholangitis in a COVID-19 patient, but there are few descriptions of imaging correlates of hepatobiliary abnormalities in COVID-19 [1,10,11,13]. Sonographic findings of gallbladder bile stasis have been reported in 54% of right upper quadrant ultrasounds in COVID-19 inpatients [1]. Specifically, whether abdominal MRI is indicated in these patients has not been examined. We are aware of only one case report of abdominal MRI being performed in a COVID-19 patient with acute pancreatitis, which reportedly did not change diagnosis from recent prior CT or elucidate a cause [11]. The objectives of this study were to quantify abdominal imaging utilization by modality and patient care setting and to determine whether abdominal MRI in COVID-19 patients at a single institution led to a change in diagnosis or management.

## 2. Materials and Methods

The Massachusetts General Hospital institutional review board approved this retrospective study. The need for informed consent was waived. A search of our institutional patient data registry was performed to identify all abdominal CT, MRI, ultrasound (US), and plain radiograph (XR) examinations in our Picture Archiving and Communication System (PACS) on adult (>18 years) patients from 15 March 2020 through 15 July 2020 who also had a positive polymerase chain reaction (PCR) test for SARS-CoV-2. This search yielded 2280 total examinations in 1122 patients. Of these, 21 examinations including 17 abdominal CTs, 3 abdominal radiographs, and 1 abdominal MRI for 15 patients were excluded for being examinations performed at outside institutions and uploaded to our PACS for secondary interpretation or comparison. The remaining 2259 abdominal imaging examinations for 1107 patients were included. Examinations were analyzed for modality type (CT, MRI, US, XR) and setting of care including inpatient, emergency, and outpatient.

Of the overall abdominal imaging cohort, MRI examinations performed on adult (>18 years) patients from 15 March 2020 through 15 July 2020 who also tested positive by PCR nasal swab assay for SARS-CoV-2 within the 100 days preceding the MRI were included for further review. One abdominal MRI was excluded because the patient tested positive for COVID-19 greater than 100 days before MRI was performed. Twelve patients were included for further review of MRI reports and medical records.

For MRI technique, all patients underwent gadoterate meglumine-enhanced (20 mL) abdominal MRI at our institution on a 1.5 or 3T scanner using a phased array multi-channel body coil. Given our multi-vendor, multi-scanner practice, exact parameters varied slightly, but representative common pre- and post-contrast sequences of the abdomen included: dynamic arterial phase 25–35 s after contrast injection, portal venous phase 70 s after contrast injection, and delayed phase 180 s after contrast injection axial T1-weighted with fat saturation, delayed 180–200 s after contrast injection coronal T1-weighted with fat saturation, axial and coronal SSFSE (single-shot fast spin echo), diffusion-weighted images, apparent diffusion coefficient map, and axial T1-weighted in and opposed phase Dixon.

Clinical MRI reports and electronic medical records for each patient were reviewed independently by a faculty abdominal radiologist with 3 years subspecialty experience and an abdominal radiology fellow (MAA, RJG). The following information was extracted from the reports and electronic medical records: number of days from positive SARS-CoV-2 PCR test to MRI examination, presence or absence of prior abdominal imaging study, prior abdominal imaging study modality, use of intravenous contrast for current MRI abdomen, patient age, gender, location (inpatient or outpatient), clinical indication and symptoms as provided by the ordering physician, clinical indication for the MRI determined by retrospective review of medical records noting if it was different from the stated indication on the study electronic requisition, presence of any positive MRI findings within the abdomen defined as those that could explain the study indications, whether MRI led to a change in management defined as whether a new diagnosis, change in therapeutic plan, or further diagnostic or therapeutic intervention based on new MRI findings occurred, and whether change in management was related or unrelated to COVID-19.

Statistical analyses included calculation of percentage of overall abdominal imaging by modality and imaging setting. Basic demographic data for patients who underwent abdominal MRI were collated using descriptive statistics. Percentages of patient cohort who had change in diagnosis and management based on MRI findings were calculated.

## 3. Results

A total of 2259 abdominal CT, MRI, US, and XR examinations were performed for 1107 COVID-19 patients (Table 1). Of 2259 abdominal imaging examinations, 1249 (55%) were XR on 394 unique patients, 694 (31%) were CT on 474 patients, 295 (13%) were US on 226 patients, and 13 (0.6%) were MRI all in unique patients (Table 1).

By imaging setting, 1799 (80%) were inpatient, 316 (14%) were emergency room, and 144 (6%) were outpatient (Table 2). Among 1249 abdominal XRs, 1216 (97%) were performed in the inpatient setting, 28 (2%) in the emergency room, and 5 (0.4%) as outpatients. Of 694 abdominal CTs, 326 (47%) were performed inpatient, 259 (37%) in the emergency room, and 109 (16%) as outpatients. For 295 abdominal or right upper quadrant USs, 246 (83%) were performed inpatient, 28 (9%) in the emergency room, and 19 (6%) as outpatients. The 13 MRI examinations were performed in 6 (46%) inpatients and 7 (54%) outpatients; none were performed in the emergency room setting (Table 2).

Out of 1107 total COVID-19 patients, abdominal MRI was performed in 12 (1.1%) within 100 days of positive SARS-CoV-2 PCR test (Table 3). The most common indications were unrelated to COVID-19 (n = 9/12, 75%). These included screening for or surveillance of malignancy (n = 4/12, 33%), evaluation of indeterminate liver lesions (n = 3/12, 25%), and surveillance of known renal mass (n = 2/12, 17%). In 25% (n = 3/12) of patients, the MRI was performed for workup of acute liver dysfunction. In 1 of 12 patients (8%), MRI resulted in a change in management which was unrelated to COVID-19 diagnosis.

Patients who underwent abdominal MRI included nine men and three women with mean age 56 years (range 33–73 years). Average time from positive SARS-CoV-2 PCR test to date of abdominal MRI was 29 days (range 1–95 days). All patients had prior abdominal imaging in our institutional PACS, including 5/12 (42%) abdominal radiograph, 7/12 (58%) abdominal ultrasound, 11/12 (92%) abdominal CT, and 5/12 (42%) abdominal MRI. At the time of abdominal MRI, six were inpatients and six were outpatients. Indications for 9/12 (75%) of MRI examinations related to follow-up or characterization of a finding unrelated to COVID-19 including screening for or surveillance of liver metastases in patients with known malignancies (n = 3), screening for hepatocellular carcinoma in a transplant liver (n = 1), follow-up of known renal masses (n = 2), and characterization of indeterminate liver lesions seen on prior ultrasound or CT (n = 3). Indications for 3/12 (25%) of MRI examinations were for investigation of cause for acute liver dysfunction including transaminitis, hyperbilirubinemia, and cholestatic laboratory abnormalities. There were no discrepancies found between the stated MRI indication on the electronic MRI requisition and the clinical question deduced by the retrospective review of the medical records preceding the MRI examination. Indications and findings for each of the 12 patients undergoing MRI are detailed in Table 3.

Of the three patients who had an MRI for workup of acute liver injury, a cause other than COVID-19 was not identified in any of them. One patient with transaminitis, hyperbilirubinemia, and elevated ALP had no MRI correlate for LFTs. Hepatosplenic iron deposition but no biliary dilation or filling defect were found in one patient with cholestatic lab abnormalities. Iron deposition in this patient was not unexpected given a history of chronic blood transfusions for renal failure associated anemia. Findings of pyelonephritis that were unchanged from prior CT was found in one patient with cholestatic lab abnormalities. All three of these patients had undergone prior abdominal ultrasound, none of which identified a cause of LFT abnormalities.

For the three patients with acute liver injury, management was not changed by MRI findings. Two of the three went on to non-focal liver biopsy 21 and 3 days after MRI for further investigation of a cause for LFT abnormalities in absence of another explanation. Pathology showed cholestatic hepatitis interpreted as possibly reflecting acute biliary obstruction, sepsis/infection, critical illness, or drug-effect in one patient, and bile duct injury and regenerative parenchymal changes as well as moderate hemosiderin accumulation within Kupffer cells interpreted to possibly be explained by ischemic or medication-induced injury or sequelae of COVID-19 infection in the second patient. A culprit drug as a cause of hepatotoxicity was not identified by the treating clinicians in the medical record in either patient. The third patient had unchanged findings of pyelonephritis compared to a recent CT for which she was continued on antibiotics that were initiated prior to MRI.

## 4. Discussion

During the first surge of COVID-19 at one institution, the most common abdominal imaging examinations were plain radiographs and CTs followed by US. MRI was rarely performed. The majority (80%) of imaging was performed in the inpatient setting. For all modalities besides MRI, inpatient imaging was most common followed by emergency room, with outpatient setting least common. For abdominal CTs, the difference between proportion of imaging performed as inpatient and emergency was smaller, with 47% inpatient and 37% emergency room. Contrary to this, although abdominal MRI outpatient exams were more common than inpatient, none were performed in the emergency room setting. Based on this data from a single institution, if abdominal imaging demand is similar, then future COVID-19 illness surges may place disproportionate demands on inpatient radiograph and CT resources compared to other abdominal imaging modalities and care settings.

These results are in line with other analyses of imaging utilization trends during the COVID-19 pandemic, which show that outpatient imaging experienced the most precipitate decline in volume relative to inpatient and emergency department examinations [21,22,23,24]. Additionally, while we found that the abdominal imaging modalities that were most commonly utilized during the first COVID-19 surge were radiographs and CTs, these same modalities have been those found to decline the least in volume at other institutions during the pandemic-related changes in radiology utilization [22,23]. Interestingly, although overall decreases in imaging volumes related to COVID-19 have been reported, in some settings, such as the emergency department, increase in per-patient imaging utilization has been reported. Our results in combination with the described published literature may be helpful in prioritizing inpatient abdominal XR and CT modality resources and taking steps to ensure COVID-19 patients can be safely imaged amidst non-COVID patients.

In our series of 12 patients who underwent abdominal MRI within 100 days of positive COVID-19 diagnosis, only 1 patient had a change in management based on the MRI findings and this was for a reason unrelated to COVID-19. In the three patients on whom MRI was performed to investigate acute liver dysfunction, a cause apart from COVID-19 was not identified, suggesting the abnormalities were likely related to COVID-19. Of note, all three of these patients had recent prior abdominal ultrasound that did not identify a non-COVID cause for LFT abnormalities such findings of chronic liver disease or cirrhotic morphology, biliary obstruction, vascular thrombosis, or hepatic masses. Therefore, in patients in whom a separate cause is not identified sonographically, MRI may not provide additional value, based on this small sample. Larger patient cohorts will be needed to more definitively evaluate the utility of abdominal MRI in COVID-19 patients.

Microvascular thrombi in multiple organs have been found in COVID-19 patients from bowel resections and autopsies and the incidence of thromboembolic events in COVID-19 patients has been found to be 35–45% compared to 5–15% of non-COVID critically ill patients [3,25,26]. Therefore, liver function abnormalities in COVID-19 may also result from microvascular coagulopathy. Additionally, since cholangiocytes and hepatocytes can express the angiotensin converting enzyme II (ACE-II) receptors by which the SARS-CoV-2 virus enters cells, direct infection may play a role in hepatic dysfunction [4]. None of these mechanistic possibilities for liver function abnormalities due to COVID-19 are likely to have MRI correlates, so the main purpose of MRI in COVID-19 patients with LFT abnormalities would be to identify a separate, treatable cause such as biliary obstruction, vascular thrombosis, or hepatobiliary masses, which would be expected to be apparent by ultrasound. Additionally, given the logistic limitations of performing abdominal MRI in critically ill patients, our findings, though limited by small sample size, do not show utility in its use in COVID-19 patients without abnormalities on abdominal ultrasound.

Existing reports of imaging in COVID-19 patients with hepatobiliary manifestations also have not demonstrated abdominal MRI to be useful. Only one case report in a patient who presented with recurring acute pancreatitis following the diagnosis of COVID-19 in the absence of other predisposing or precipitating factors has reported abdominal MRI findings. In this case, the diagnosis was made by symptoms, laboratory abnormalities, and CT findings after an unremarkable US. Subsequent abdominal MRI with MRCP did not reveal a change or separate cause for pancreatitis [11]. Additional reports of hepatobiliary manifestations of COVID-19 include one patient with obstructive cholangitis identified by CT and ERCP, but this may have been mechanical, related to stones and sludge rather than directly to COVID-19 given the endoscopic description of findings and the fact that a SARS-CoV-2 PCR positive test occurred 11 days after ERCP and biliary obstruction relief [10]. Acute hepatitis, rapidly progressive liver failure, and hepatic encephalopathy in absence of another viral, alcohol, drug-related, or other cause have also been reported as the presentations of COVID-19 in patients whose abdominal ultrasounds showed no biliary dilation, hepatic vascular thrombosis, or other cause [5,8,9]. Reported positive imaging hepatobiliary findings in COVID-19 patients with GI manifestations include nonspecific cholestasis, gallbladder thickening, and pericholecystic fluid, which could be seen in primary gallbladder inflammation or as reactive changes to many causes of hepatic dysfunction [1,5].

Limitations of our study include its single-center, retrospective nature, which reduces generalizability and could introduce selection bias. It is unclear whether all diagnoses considered positive were COVID-19 related; some findings may be the result of pre-existing conditions unrelated to COVID-19 or to critical illness in general. The portion of our study examining utility of abdominal MRI included only a small number of cases, but we felt a rapid analysis of this new and evolving patient population, in order to guide ongoing management decisions, outweighed waiting for further study accrual and delaying reporting of findings. Another limitation is that MRI findings were extracted from clinical reports rather than reassessed under experimental conditions. However, this approach better reflects real-life imaging interpretation and so may be more applicable to clinical practice.

## 5. Conclusions

During the first surge of COVID-19 at one institution, the most common abdominal imaging examinations were XRs and CTs followed by US. The majority (80%) of imaging was performed in the inpatient setting. Future COVID-19 illness surges may place disproportionate demands on inpatient XR and CT resources compared to other abdominal imaging modalities and care settings. Abdominal MRI was rarely performed and was found to change diagnosis or management in only one patient, for a condition unrelated to COVID-19.

## Figures and Tables

**Table 1:** Abdominal imaging usage in COVID-19 patients by modality.

	All Modalities	Abdominal XRs (% Total)	Abdominal CTs (% Total)	Abdominal USs (% Total)	Abdominal MRIs (% Total)
Exams	2259	1249 (55%)	694 (31%)	295 (13%)	13 (0.6%)
Patients	1107	394 (36%)	474 (43%)	226 (20%)	13 (1%)

**Table 2:** Abdominal imaging examinations in COVID-19 patients by care setting.

	All Modalities	Abdominal XRs (% Total)	Abdominal CTs (% Total)	Abdominal USs (% Total)	Abdominal MRIs (% Total)
Total	2259	1249	694	295	13
Inpatient	1799 (80%)	1216 (97%)	326 (47%)	246 (83%)	6 (46%)
Emergency	316 (14%)	28 (2%)	259 (37%)	28 (9%)	0 (0%)
Outpatient	144 (6%)	5 (0.4%)	109 (16%)	19 (6%)	7 (54%)

**Table 3:** Indications and findings from MRI examinations in COVID-19 patients.

Patient	MRI Indication	Findings	Change in Diagnosis/ Management
1	LFT abnormalities	No correlate for LFT abnormalities	No
2	Screening for metastases	No abdominal metastases	No
3	LFT abnormalities	Hepatosplenic iron deposition No biliary obstruction	No
4	Renal mass surveillance	Enlarging renal mass	No
5	Indeterminate liver lesion on chest CT	Hemangiomas	No
6	Indeterminate liver lesion on abdominal US	No correlate to US finding	No
7	Renal mass surveillance	Unchanged renal masses	No
8	Liver metastases surveillance	Slightly enlarging liver metastases	No
9	Indeterminate liver lesion on chest CT	Liver and bone metastases	Yes: new liver metastases treated with stereotactic radiation
10	Screening for hepatocellular carcinoma in liver transplant	No focal liver lesions	No
11	LFT abnormalities	Unchanged pyelonephritis and associated abscess	No
12	Staging of known hepatocellular carcinoma	Bilobar hepatocellular carcinoma	No

## Data Availability

Apart from the data described and available in tabulated form in this article, no other data were created or analyzed in this study.
